# From the Uncharacterized Protein Family 0016 to the GDT1 family: Molecular insights into a newly-characterized family of cation secondary transporters

**DOI:** 10.15698/mic2020.08.725

**Published:** 2020-06-15

**Authors:** Louise Thines, Jiri Stribny, Pierre Morsomme

**Affiliations:** 1 Louvain Institute of Biomolecular Science and Technology, UCLouvain, Louvain-la-Neuve, Belgium.

**Keywords:** UPF0016 family, Gdt1 family, Secondary transporters, Glycosylation, Calcium, Manganese, Gdt1p, TMEM165

## Abstract

The Uncharacterized Protein Family 0016 (UPF0016) gathers poorly studied membrane proteins well conserved through evolution that possess one or two copies of the consensus motif Glu-x-Gly-Asp-(Arg/Lys)-(Ser/Thr). Members are found in many eukaryotes, bacteria and archaea. The interest for this protein family arose in 2012 when its human member TMEM165 was linked to the occurrence of Congenital Disorders of Glycosylation (CDGs) when harbouring specific mutations. Study of the UPF0016 family is undergone through the characterization of the bacterium *Vibrio cholerae* (MneA), cyanobacterium *Synechocystis* (SynPAM71), yeast *Saccharomyces cerevisiae* (Gdt1p), plant *Arabidopsis thaliana* (PAM71 and CMT1), and human (TMEM165) members. These proteins have all been identified as transporters of cations, more precisely of Mn^2+^, with an extra reported function in Ca^2+^ and/or H^+^ transport for some of them. Apart from glycosylation in humans, the UPF0016 members are required for lactation in humans, photosynthesis in plants and cyanobacteria, Ca^2+^ signaling in yeast, and Mn^2+^ homeostasis in the five aforementioned species. The requirement of the UPF0016 members for key physiological processes most likely derives from their transport activity at the Golgi membrane in human and yeast, the chloroplasts membranes in plants, the thylakoid and plasma membranes in cyanobacteria, and the cell membrane in bacteria. In the light of these studies on various UPF0016 members, this family is not considered as uncharacterized anymore and has been renamed the Gdt1 family according to the name of its *S. cerevisiae* member. This review aims at assembling and confronting the current knowledge in order to identify shared and distinct features in terms of transported molecules, mode of action, structure, etc., as well as to better understand their corresponding physiological roles.

## INTRODUCTION

Proteins are classified into families in which members share a common evolutionary origin, reflecting their related functions and similarities in terms of sequence and/or structure. Therefore, when a novel protein is identified, its functional properties are often hypothesized based on the functional features of the group it is predicted to belong to. The number of defined protein families increased drastically with the development of genome sequencing. However, some of these families have absolutely no assigned function, for none of their members; these are the so-called Uncharacterized Protein Families (UPFs). This review focuses on one of these UPFs: the UPF0016 family (Pfam PF01169, TCDB number: 2.A.106). This protein family was described in 2014 as gathering membrane proteins found in many eukaryotes, bacteria, and archaea [1]. Numerous paralogs are mainly found in plants, which possess UPF0016 members in various subcellular compartments, including the Golgi [2], endoplasmic reticulum (ER) [2], and chloroplasts [3–7], whereas the non-plant eukaryotic UPF0016 members were all found at the Golgi up to now [8, 9]. All UPF0016 members are defined by the presence of one or two copies of the Glu-ϕ-Gly-Asp-(Arg/Lys)-(Ser/Thr) consensus motif (with ϕ being any hydrophobic residue) [1]. Despite the high level of conservation within this protein family, some protein sub-groups share specific features in terms of length of the N-terminal part or enrichment in negatively charged residues in specific regions for instance [1]. From a broader view, the UPF0016 family belongs to the LysE superfamily that gathers eleven families of transport proteins that catalyze export of amino acids, lipids, and heavy metal ions [10].

**Figure 1:**
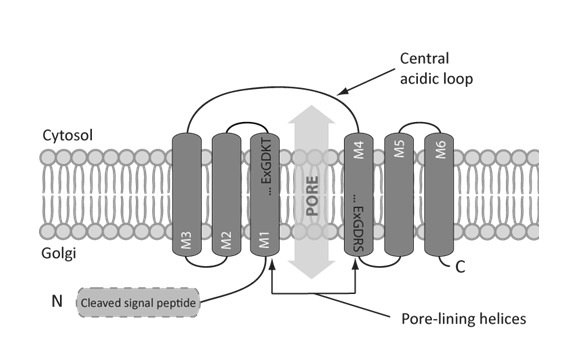
FIGURE 1: Predicted topology of the eukaryotic UPF0016 members. The eukaryotic UPF0016 members are predicted to consist of two clusters of three transmembrane spans assembled in an antiparallel orientation and separated by a central loop enriched in negatively charged residues found at the cytosolic side in yeast, human, and bacteria. The featured UPF0016 motif Glu-ϕ-Gly-Asp-(Arg/Lys)-(Ser/Thr) is found in two copies, in the transmembrane spans one and four. Due to the hydrophilic nature of the residues found in this motif, and its localization in hydrophobic transmembrane spans, their two copies were hypothesized to form the pore of the protein through which the transported ions would cross the membrane [1].

Based on *in silico* approaches, the predicted topology of the eukaryotic UPF0016 members consists of two homologous clusters of three transmembrane spans with an opposite orientation in the membrane. These two clusters are separated by a central loop enriched in acidic amino acids (**Figure 1**) [1]. Interestingly, the predicted topology of the eukaryotic UPF0016 members is similar to that encountered in most of the families that belong to the LysE superfamily, mainly in terms of the presence of two internal repeats of three transmembrane spans [10]. More diversity in terms of topology can be observed among the prokaryotic members of the UPF0016 family. Indeed, some prokaryotes possess one gene coding for one UPF0016 member of three transmembrane spans that is predicted to auto-assemble as a homodimer while others possess two adjacent genes, encoding two different UPF0016 members of three transmembrane spans that are hypothesized to assemble as heterodimers. Finally, the eukaryotic-like topology, with six transmembrane spans, is also found among prokaryotes [1].

When comparing the protein sequences of numerous UPF0016 members, the most conserved regions are the transmembrane spans and the featured motif Glu-ϕ-Gly-Asp-(Arg/Lys)-(Ser/Thr). This latter is predicted to localize in the transmembrane spans one and four of the eukaryotic members (**Figure 1**) [1]. Despite its hydrophobic environment, this motif contains two negatively charged (Asp and Glu), one positively charged (Arg or Lys), and one hydroxyl-containing (Ser or Thr) residue. Based on the role of similar residues in coordinating transported cations for proteins with available resolved structure [11], the two copies of the UPF0016 featured motif were hypothesized to form the pore of the putative transporter.

Since 2012 and the establishment of a causal link between the presence of mutations within TMEM165 (Transmembrane protein 165) and the occurrence of congenital disorders of glycosylation (CDGs) [8], many studies have been conducted on the functional properties of bacterial, yeast, plant, and human UPF0016 members. These studies reported a role of the UPF0016 members mainly in cation transport, thereby influencing essential processes like protein glycosylation in yeast and human, lactation in human, photosynthesis in plants and cyanobacteria, and Mn^2+^ toxicity in bacteria. This review aims at gathering the state-of-the-art knowledge accumulated on various UPF0016 members, thereby clarifying their molecular function in various organisms, and their subsequent implication in cellular processes. The mode of action (direction of transport, cations transported, structure-function relationship, etc.) of the UPF0016 members is also discussed in this review to shed light on conserved aspects but also on key differences that seem to occur within the family in terms of the aspects aforementioned, since this could be determinants of their physiological implication.

## THE UPF0016 FAMILY AND CATION TRANSPORT

As previously stated, the UPF0016 family belongs to the LysE superfamily that gathers transporters of amino acids, lipids, and heavy metal ions [10]. In addition, similarities in terms of predicted topology of the UPF0016 members on the one hand, and of members of the cation-Ca^2+^ exchangers superfamily, that gathers transporters of Ca^2+^ against its electrochemical gradient by utilizing the downhill gradients of other cation species like H^+^, Na^+^, or K^+^ [12], on the other hand, have also been observed. Based on these observations, the UPF0016 members were hypothesized to act as secondary cation transporters [1]. Since then, and as detailed below, several studies indicated transport of Mn^2+^ by all studied UPF0016 members, and of Ca^2+^ and/or protons by some of them.

### Transport of Mn^2+^ by UPF0016 members

Since 2016, studies on the role of the six characterized UPF0016 members (MneA, SynPAM71, Gdt1p, PAM71, CMT1, and TMEM165) in Mn^2+^ homeostasis started to appear in the literature. In the plant *Arabidopsis thaliana*, the paralogs PAM71 (Photosynthesis-affected mutant 71, also called CCHA1, for Chloroplast Ca^2+^/H^+^ antiporter, or BICAT1, for Bivalent cation transporter 1) and CMT1 (Chloroplast Mn^2+^ transporter 1, also called BICAT2, for Bivalent cation transporter 2, or PAM71-HL) were both identified as involved in Mn^2+^ homeostasis from their localization at the thylakoid membrane [3–5] and inner envelope [4, 6, 7] of the chloroplasts, respectively. At the plant level, the two corresponding mutant plants show affected photosynthesis [3–7], reduced starch synthesis [4–7] and chlorophyll content [3, 4, 7], and decreased growth rate [3–7]. Interestingly, in both cases, the photosynthetic deficiency of the mutant plants could be partially suppressed in case of addition of Mn^2+^ to the growth medium [5–7]. Besides, these two chloroplast proteins are required for proper function and abundance of the Mn^2+^-containing oxygen-evolving complex of the photosystem II (PSII) (Mn_4_CaO_5_) [3, 5–7] and both mutant plants show reduced incorporation of Mn^2+^ per unit of PSII [5, 6]. Hence, these data all indicate a correlation between the photosynthetic deficiencies of the mutant plants and a reduced Mn^2+^ content within the chloroplasts. To further confirm their cation transport ability, *CMT1* or *PAM71* were individually expressed in the yeast strain *pmr1*Δ, devoid of its Golgi Ca^2+^-Mn^2+^ ATPase. This led in both cases to the suppression of the well-established Mn^2+^ sensitivity of *pmr1*Δ [5, 6]. Expression of *CMT1* in yeast further suppresses the growth defect observed under Mn^2+^-limited environment of the *smf1*Δ strain, devoid of its Mn^2+^ importer, through a re-increased cellular Mn^2+^ pool, again strengthening its ability to transport Mn^2+^ [7]. Interestingly, the double mutant CMT1-PAM71 shows a phenotype closer to the CMT1 than that of the PAM71 mutant, thereby illustrating that CMT1 is the limiting step in Mn^2+^ delivery to the chloroplast. This means that CMT1 would function upstream of PAM71 for Mn^2+^ uptake over the chloroplast envelope and then the thylakoid membrane, respectively [6]. Taken together, these studies demonstrate the key role of both CMT1 and PAM71 in chloroplast development and Mn^2+^ supply to the PSII, which are both crucial for proper photosynthesis.

Similar to the observations in plants, the UPF0016 ortholog found in the cyanobacterium *Synechocystis*, SynPAM71 (also called Mnx, for Mn^2+^ exporter), is also crucial for maintenance of Mn^2+^ homeostasis and proper photosynthesis. The subcellular localization of SynPAM71 is not clearly established yet: Gandini *et al.* report localization both at the plasma membrane and, to a lesser extent, at the thylakoid membranes [13] whereas Brandenburg *et al.* localize SynPAM71 only at the thylakoid membranes [14]. The SynPAM71 loss-of-function line displays intracellular Mn^2+^ enrichment (particularly in the thylakoid lumen), reduced levels of chlorophyll, as well as reduced abundance of the PSII and defects in its photochemistry [13, 14]. Interestingly, while Mn^2+^ supplementation to the external medium improves photosynthesis in the mutant plants, the cyanobacterial mutant shows a Mn^2+^-sensitive phenotype [13, 14]. Additionally, the SynPAM71 mutant is not able to release previously-internalized radioactive ^54^Mn^2+^ from its internal pools, thereby suggesting toxic Mn^2+^ accumulation in this SynPAM71 loss-of-function line [14]. The transport ability of SynPAM71 was further supported by the fact that its expression in yeast also suppresses the Mn^2+^-sensitive phenotype of the *pmr1*Δ strain [14]. Taken together, these data all suggest a role of the *Synechocystis* UPF0016 member in Mn^2+^ export to prevent Mn^2+^ toxicity that could in turn impair essential processes like photosynthesis. Interestingly, similar effects on the photosynthetic efficiency were also attributed to the *Chlamydomonas* UPF0016 mutant GLD1 (Glucose-6-phosphate-1-dehydrogenase), thereby indicating a conserved function of the UPF0016 members from plants, algae, and cyanobacteria, in photosynthesis through regulation of the cation internal pools [5].

Recently, the MneA (Mn^2+^ exporter A) UPF0016 member from the bacterium *Vibrio cholerae,* a pathogenic gram-negative bacterium, was identified as a putative Mn^2+^ exporter. Indeed, the MneA mutant exhibits sensitivity to high Mn^2+^ concentration and shows an increased intracellular Mn^2+^ level when exposed to Mn^2+^, compared to a wild-type strain [15]. Due to the role of Mn^2+^ as antioxidant, the MneA mutant also shows increased resistance to H_2_O_2_ compared to the wild-type through internal accumulation of Mn^2+^ [15]. Besides, expression of *mneA* suppresses the Mn^2+^-sensitive phenotype of an *Escherichia coli* strain carrying a mutation in its Mn^2+^ export gene *mntP* and decreases its Mn^2+^ content [15, 16], thereby showing the function of MneA in Mn^2+^ export.

Last but not least, evidence regarding the involvement of the human UPF0016 member TMEM165 in Mn^2+^ homeostasis has also been made available. First, the abundance of a Golgi-localized protein whose stability is known to be Mn^2+^-sensitive, GPP130, is altered in TMEM165-depleted cells, thereby indicating a disturbed Mn^2+^ homeostasis at the Golgi level [17]. Besides, TMEM165 itself is rapidly and specifically degraded in lysosomes in response to excess of Mn^2+^ in the extracellular medium [18]. Glycosylation defects are known to occur in TMEM165-depleted cells (as will be further described below). Interestingly, addition of Mn^2+^ to the extracellular medium restores glycosylation in TMEM165-depleted cells [17]. In *Saccharomyces cerevisiae*, addition of Mn^2+^ to the yeast growth medium both triggers degradation of its Golgi-localized UPF0016 member Gdt1p (Gcr1-dependent translation factor) and suppresses the glycosylation defects that can be observed in *gdt1*Δ cells cultured in the presence of Ca^2+^ excess [17, 19]. Besides, Gdt1p is involved in resistance to high Mn^2+^ concentrations, controls the cellular Mn^2+^ pools, and modulates the enzymatic activity of Sod2p, an enzyme that requires Mn^2+^ as cofactor for proper activity [20]. Interestingly, production of a truncated version of TMEM165 (lacking its first 78 N-terminal amino acids) in the yeast strain *gdt1*Δ restores its Mn^2+^ sensitivity and cellular Mn^2+^ stores, thereby illustrating a conserved function in Mn^2+^ homeostasis [21]. The transport activity of both TMEM165 (produced again with truncated 78 N-terminal amino acids) and Gdt1p was further demonstrated by producing these proteins in Fura-2-loaded *Lactococcus lactis* cells, in which a Mn^2+^-induced Gdt1p-dependent quenching of the fluorescence emitted by Fura-2 can be observed [20, 21]. Taken together, these data clearly illustrate the key role of the UPF0016 members from various species in Mn^2+^ homeostasis.

### Transport of Ca^2+^ by UPF0016 members

Apart from their role in Mn^2+^ homeostasis, the yeast, plant, and human UPF0016 members were suggested to be involved in Ca^2+^ homeostasis. Most of the evidence of the involvement of the UPF0016 members in Ca^2+^ homeostasis actually arises from studies conducted on the *S. cerevisiae* member Gdt1p. First of all, growth of the *gdt1*Δ strain is reduced in the presence of high external Ca^2+^ concentration (750 mM), compared to the wild-type, illustrating its role in coping with Ca^2+^ stress [9]. Interestingly, this increased sensitivity of the *gdt1*Δ strain towards Ca^2+^ could be suppressed when expressing (i) a truncated version of the human ortholog *TMEM165* (lacking its first 78 N-terminal residues [21]), (ii) a truncated version of the *A. thaliana* UPF0016 members *PAM71* (lacking its first 155 N-terminal residues [3]) or *CMT1* (lacking its first 131 N-terminal residues [4]), or (iii) the *Candida albicans* ortholog *CaGDT1* [22], thereby suggesting a conserved function in Ca^2+^ homeostasis. Another indication that Gdt1p is involved in Ca^2+^ homeostasis lies in its established interaction with *PMR1* at the genetic level. *PMR1* encodes a well-characterized yeast Ca^2+^-Mn^2+^ P-type ATPase colocalizing with Gdt1p at the cis- and medial-Golgi. This genetic interaction is reflected by the fact that *gdt1*Δ*pmr1*Δ is more sensitive to Ca^2+^ than both single mutants [19] and that *GDT1* expression level and activity depends on *PMR1* expression and ability to transport Ca^2+^ and/or Mn^2+^ [23], thereby suggesting similar functions for these two proteins. Besides, the level of expression of *GDT1* influences the cellular Ca^2+^ accumulation (most likely since Gdt1p modulates the intraluminal Golgi cation content and cations can exit the cell from the Golgi through secretory vesicles trafficking) as well as the Ca^2+^ response observed after exposure of yeast cells to a salt stress [19], all supporting its role in yeast Ca^2+^ homeostasis. In the yeast *C. albicans*, the comparison of the double deletion strain to each of the single deletions indicates that CaGdt1p and CaPmr1p also interact at the genetic level. Indeed, further deletion of *CaGDT1* in a strain deleted for *CaPMR1* increases (i) its sensitivity towards cell wall and ER stresses, (ii) its ability to accumulate radioactive ^45^Ca^2+^, and (iii) its hypersensitivity to inhibitors of the Ca^2+^-mediated calcineurin signaling pathway [24]. Transcriptomic analyses further revealed that CaGdt1p is involved in the regulation of cellular transport of metal ions and amino acids [24]. In this pathogen, CaGdt1p also interacts at the genetic level with the high-affinity plasma membrane Ca^2+^ channel CaCch1p/CaMid1p, again strengthening the role of CaGdt1p in Ca^2+^ homeostasis. Indeed, extra deletion of *CaGDT1* in a strain deleted for *CaCCH1* or *CaMID1* suppresses its sensitivity to cold stress, but also increases its sensitivity to antifungal drugs [22].

Whole-cell patch-clamp analyses on HeLa cells overexpressing *TMEM165* further enabled to assign a cation transport activity to TMEM165 [9]. Interestingly, the membrane currents that were observed for the *TMEM165*-expressing HeLa cells were decreased in the presence of the Ca^2+^-chelating agent EGTA, suggesting Ca^2+^ transport by TMEM165 [9]. The use of the Ca^2+^-sensitive fluorescent probe Fura-2 in HeLa cells overexpressing *TMEM165* further supported the role of this human protein in Ca^2+^ homeostasis [9]. Like in yeast, TMEM165 genetically interacts with the human ortholog of Pmr1p, the Golgi Ca^2+^-Mn^2+^ ATPase SPCA1, in the way that the abundance of TMEM165 depends on the abundance and function of SPCA1. The authors of this study even suggest that SPCA1 and TMEM165 physically interact [25]. Interestingly, in mice, the level of expression of *TMEM165* in lactating mammary tissues increases by a 25-fold factor during lactation while forced cessation of lactation leads to a rapid decrease of its expression. As about 40% of Ca^2+^ in milk (30-80 mM) is thought to be first stored in the Golgi lumen of the mammary epithelial cells to be then secreted into milk, both timing and magnitude of expression of *TMEM165* place this protein as a potential contributor to mammary Golgi Ca^2+^ transport needs [26].

Finally, the two plant UPF0016 members from the *A. thaliana* chloroplasts, PAM71 and CMT1, were also linked to Ca^2+^ homeostasis, although their ability to transport Ca^2+^ cations still remains under debate. The identification of these two plant proteins as putative Ca^2+^ transporters arises from decreased radioactive ^45^Ca^2+^ uptake in isolated thylakoids and chloroplasts of the PAM71 and CMT1 mutant plants, respectively [4]. Besides, while modulation of the Ca^2+^ concentration in the chloroplast stroma is known to occur in the transition between light and dark phases of photosynthesis, this [Ca^2+^]_stroma_ was detected as affected in the two mutant plants compared to the wild-type, thereby reflecting an influence on Ca^2+^ signaling [4]. In the light of the key role of Ca^2+^ in photosynthesis, both in terms of signaling and regulation of involved enzymes, the previously mentioned defects in terms of growth and photosynthesis observed in the two mutant plants could therefore partly be due to disturbed Ca^2+^ partitioning in the chloroplasts [3, 4]. Further study of the thylakoid membrane PAM71 mutant plant shows that it is sensitive to high external Ca^2+^ concentrations and EGTA [3], and that it has a modified cytosolic Ca^2+^ content compared to the wild-type [3]. However, contradictory data regarding the role of the *A. thaliana* UPF0016 members in Ca^2+^ homeostasis are available: whereas Schneider *et al.* reported a higher Ca^2+^ uptake in the thylakoids of PAM71 mutants [5], Frank *et al.* observed a decrease in such Ca^2+^ uptake [4]. In addition, Frank *et al.* notified differences in terms of chloroplast Ca^2+^ content between wild-type and CMT1 mutant plants [4] whereas no such differences were observed by Zhang *et al* [7]. These contradictions reveal that further studies are needed to clearly evaluate the role of PAM71 and CMT1 in Ca^2+^ homeostasis, and to determine whether their impact on photosynthesis is of primary or secondary nature.

Recently, the Ca^2+^ transport activity of the yeast Gdt1p [19], the plant PAM71 [4], and the human TMEM165 [21] was further supported by the recording of a more pronounced Ca^2+^ influx in bacteria producing one of these UPF0016 members with the Ca^2+^-sensitive fluorescent probe Fura-2, compared to control cells containing an empty plasmid. Taken together, these data suggest that the Golgi UPF0016 members from human and yeast, and the chloroplast UPF0016 members from plants influence the cellular Ca^2+^ distribution through their transport ability.

At that stage, it is interesting to point out that, while Gdt1p, TMEM165, PAM71, and CMT1 have all been linked to both Ca^2+^ and Mn^2+^, there is no current indication of Ca^2+^ transport by the bacterial SynPAM71 and MneA. This might suggest that, within the UPF0016 protein family, some members could transport the two cations while others would transport only one or the other. Of course, this statement has to be confirmed by further biochemical characterization of these UPF0016 members.

### Transport of protons by UPF0016 members

The UPF0016 members are hypothesized to work as secondary transporters. To date, few pieces of evidence indicate that the UPF0016 members would exchange Ca^2+^ and/or Mn^2+^ against H^+^ and would thereby be involved in pH homeostasis. In this context, Demaegd *et al.* first noticed a decreased lysosomal pH in both fibroblasts of TMEM165-deficient patients and TMEM165-depleted HeLa cells, in comparison with control individuals or cells [9]. More recently, the use of an *in situ* Golgi-localized pH-sensitive probe enabled to highlight acidification of the Golgi apparatus in the absence of TMEM165 [27]. In the same line, the *A. thaliana* PAM71 mutant plants show a pH-sensitive phenotype and an increased cytoplasmic pH, which is thought to reflect disturbed stromal pH whose proper maintenance is of importance for effective photosynthesis [3]. Nevertheless, a reduced H^+^-ATPase activity was also observed in PAM71 mutant plants [3,5]. Therefore, one cannot exclude that the effects of PAM71 on chloroplast pH homeostasis are of secondary nature rather than directly linked to H^+^ transport. In yeast, the Ca^2+^ transport activity of Gdt1p depends on the pH gradient across the Golgi membrane created by the Golgi proton V-ATPase. More precisely, Gdt1p seems to promote Ca^2+^ sequestration within the Golgi lumen when this organelle is correctly acidified by the V-ATPase, but works in the opposite direction, from the Golgi to the cytosol, when such acidification is disrupted by deleting the gene coding for the V-ATPase [28]. Interestingly, the Ca^2+^ influx observed in Fura-2-loaded *L. lactis* cells producing Gdt1p depends on the external pH in the way that the higher the external pH, the higher the influx rate, being consistent with the model of cation/proton antiporter [19]. Finally, since proton is known to be a by-product of all glycosylation reactions, including during milk production in the Golgi lumen of lactating mammary cells, the increased expression of *TMEM165* during lactating periods may contribute to deacidify the Golgi lumen by transporting the accumulated protons to the cytosol in exchange of Mn^2+^ and/or Ca^2+^ [26].

While these data all suggest a role of the human TMEM165, the yeast Gdt1p, and the *A. thaliana* PAM71 in pH homeostasis, there is still a lack of direct H^+^ transport evidence for the UPF0016 members. Therefore, to date, one cannot exclude that the altered pH homeostasis observed in case of disruption of the UPF0016 members could be a secondary consequence of altered Ca^2+^ and/or Mn^2+^ homeostasis.

### Combining transport of Ca^2+^, Mn^2+^, and H^+^…

**Figure 2:**
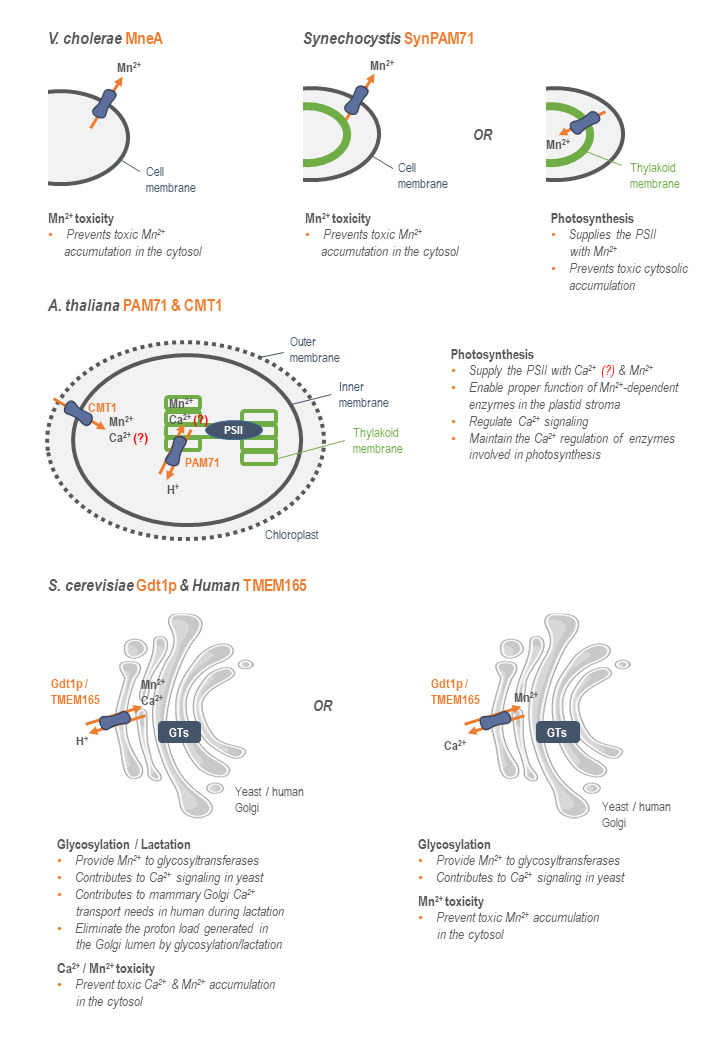
FIGURE 2: Mode of action of the currently studied UPF0016 members. The direction of transport of the UPF0016 members' substrates from their respective subcellular localization is indicated by orange arrows. Their putative implication in key physiological processes is indicated. Functions that are still unclear in the literature are indicated by *(?)* (PSII: photosystem II, GTs: glycosyltransferases).

When combining the data aforementioned, it appears that bacterial, yeast, plant, and human UPF0016 members all transport Ca^2+^, Mn^2+^, and/or H^+^ as secondary transporters. **Figure 2** illustrates the putative mechanisms of action of the studied UPF0016 members according to the biochemical data accumulated to now. Mainly, there is indirect evidence of transport of these three cations for the yeast Gdt1p and the human TMEM165. One of the possible mechanisms of transport with these three actors consists of a Ca^2+^-Mn^2+^/H^+^ antiporter, where Ca^2+^ and Mn^2+^ would be transported at the Golgi level in one direction in exchange of protons. However, and in accordance with the limited and relatively indirect evidence of transport of protons, it was suggested that these UPF0016 members would work as Ca^2+^/Mn^2+^ antiporters [23, 29]. In *A. thaliana*, data from different studies led to the conclusion that Mn^2+^ cations and, if transported, Ca^2+^ cations would also be transported in the same direction, towards the chloroplast lumen [3–7], with indication of transport of protons only for PAM71 [3]. In cyanobacteria (SynPAM71) and bacteria (MneA), there is up to now only indication of Mn^2+^ transport. If working as secondary transporters, as suspected from their predicted topology, the nature of the counter-ion still remains to be identified. In other words, combination of the published data suggests putative differential specificity among the UPF0016 members. Further study of the substrate specificity of a wide range of UPF0016 members would answer this question and putatively lead to the establishment of a correlation between the substrate specificity of the UPF0016 members and evolution.

Another missing piece of the puzzle is the direction of transport of these cations. Nevertheless, based on data interpretation, the human and yeast orthologs TMEM165 and Gdt1p could transport Ca^2+^ and Mn^2+^ from the cytosol to the Golgi lumen in exchange of protons from the Golgi to the cytosol, if working as Ca^2+^-Mn^2+^/H^+^ antiporters. In case of a Ca^2+^/Mn^2+^ antiporter, data suggest that Mn^2+^ would be imported in the Golgi in exchange of Ca^2+^ [23, 29]. In plants, Mn^2+^ and, if transported, Ca^2+^ would be transported from the cytosol to the stroma via CMT1, and then from the stroma to the thylakoid lumen via PAM71 [3–7]. In cyanobacteria, Mn^2+^ would be transported from the cytosol to the thylakoid lumen and/or the extracellular medium, depending on the considered subcellular localization of SynPAM71 [13, 14]. Finally, the bacterial UPF0016 members are suspected as transporting Mn^2+^ from the cytosol to the extracellular medium [15, 16], with no identified counter-ion. However, one should keep in mind that these directions of transport aforementioned are highly speculative and that the UPF0016 members, like numerous secondary transporters, could work reversely.

## TRANSCRIPTIONAL AND TRANSLATIONAL REGULATION OF THE UPF0016 MEMBERS

In the light of their cation transport activity, it would not be surprising that the UPF0016 members would be regulated at the transcriptional and/or translational levels according to the concentrations of the transported substrates. In bacteria, 328 out of 333 screened strains that possess a UPF0016 member have the corresponding gene under the control of the Mn^2+^-regulated riboswitch yybP-ykoY, thereby indicating a transcriptional Mn^2+^-dependent regulation [16, 30]. Additionally, five bacterial UPF0016 members (from the cyanobacterium *Anabaena* and four halobacteria) are under the control of the two-component system ManS (histidine kinase that senses the extracellular concentration of Mn^2+^)/ManR (DNA-binding response regulator that regulates expression of targeted genes) [31–33] that would modulate the expression of the UPF0016 members according to the neighboring Mn^2+^ concentration [34, 35]. More direct expression analyses show that expression of the bacterial *V. cholerae mneA* is induced after addition of Mn^2+^ to the extracellular medium [15], while *CMT1* expression in plant is reduced in the presence of Mn^2+^ excess [6]. Besides, the protein abundance of both Gdt1p and TMEM165 is reduced in case of external Mn^2+^ excess [18]. Although highly speculative, one might suggest based on these data that the presence of Mn^2+^ excess triggers (i) increased abundance of the bacterial UPF0016 members to detoxify the cytosol through their export activity, and (ii) decreased abundance of the eukaryotic UPF0016 members to prevent toxic Mn^2+^ excess in the lumen of the organelle this cation is sent to.

The activity of the yeast Gdt1p is reported to be negatively regulated by the Ca^2+^-induced calcineurin pathway [28]. No more is known regarding the Ca^2+^-dependent regulation of the UPF0016 members. This aspect should therefore be addressed in the near future.

## THE UPF0016 FAMILY AND GLYCOSYLATION

Specific mutations in the gene coding for the human Golgi-localized UPF0016 member TMEM165 were identified as causing a sub-type of CDGs [8, 36]. Since the scientific interest for the UPF0016 family mainly arises from this fact, this section gathers the up-to-date information that connects the family members to this essential cellular process. CDGs refer to a family of rare inherited diseases resulting in defects in the synthesis of glycans and in their attachment to proteins or lipids [37]. At that time, since the function of TMEM165 was undefined, the causal link between the presence of these mutations and the occurrence of the pathology was not fully understood. To date, five specific mutations within the gene coding for TMEM165 (four missense mutations: ^126^Arg>His, ^126^Arg>Cys, ^304^Gly>Arg, and ^108^Glu>Gly) and one mutation that activates a cryptic splice site (c.792+182 G>A)), identified in six patients, are reported to cause CDGs [8, 36]. With more than 50% of eukaryotic proteins being glycosylated, it is not surprising that TMEM165-CDG patients show various and severe symptoms like growth and psychomotor retardation, muscular weakness, skeletal dysplasia, fat excess, and fever episodes among others, resulting in decreased life expectancy [8, 36, 38] (reviewed in [39]).

At the molecular level, patients suffering from TMEM165-CDGs, as well as TMEM165-depleted HeLa and HEK cells, show affected protein glycosylation profile, mainly in terms of relative hypo-sialylated and hypo-galactosylated N-glycans [8, 17]. Interestingly, while these abnormalities point to defects in glycans synthesis at the Golgi, the Golgi network was found dilated and fragmented in affected individuals [8]. Besides, the glycosylation defects observed in culture cell lines could be suppressed by supplementing the growth medium with Mn^2+^ [17], as previously stated, but also with galactose [40]. According to this observation, and based on the strong protein hypo-galactosylation in TMEM165-CDG patients, oral galactose supplementation was considered as a treatment of TMEM165-CDGs and has now proven to improve clinical and biochemical parameters of the patients [40]. Antisense RNA therapy targeting TMEM165 mRNAs was also successfully considered in case of pathogenic splicing (mutation c.792+182 G>A) [41].

The role of Gdt1p, the *S. cerevisiae* Golgi-localized UPF0016 member, in glycosylation efficiency has also been investigated. Surprisingly, no glycosylation deficiency could be detected in the strain deleted for *GDT1*. Protein glycosylation is however affected when growing the *gdt1*Δ strain in a Ca^2+^-rich medium (500 mM) [17, 19]. Similar to observations in human cells, glycosylation is restored upon the additional presence of Mn^2+^ cations [17, 19]. Structural analysis of the yeast glycans, mainly constituted of polymannan chains, further revealed that the *gdt1*Δ mutant cultured in presence of high Ca^2+^ concentrations presents strong late Golgi glycosylation defects with a lack of α-1,2 mannoses substitution and α-1,3 mannoses termination [23].

Finally, it was shown in zebrafish that inhibition of *TMEM165* expression in developing embryos causes altered initiation, processing, and extension of N-glycans, together with altered cartilage and bone development [42]. Taken together, these data indicate that, despite not being identified as direct actors in glycosylation, the UPF0016 members are crucial for proper protein glycosylation.

## FROM ALTERED UPF0016 TRANSPORT FUNCTION TO DISTURBED PHYSIOLOGICAL PROCESSES

The previous sections clearly illustrate that the UPF0016 members influence the cellular cation distribution, most likely through their common ability to transport Mn^2+^ but also Ca^2+^ and/or H^+^ for some of them. Disruption of the ionic intracellular pools in case of malfunction of the UPF0016 members might in turn influence essential physiological processes in their respective organism, as further detailed in this section. Of course, while focused on the UPF0016 members, it is important to mention that these transporters most likely act in concert with other transporters from other protein families that are also essential for proper cation homeostasis.

The link between TMEM165 and glycosylation can first be examined in the light of its transport activity. Many glycosyltransferases involved in glycosylation actually require interaction with Mn^2+^ as cofactor for proper activity. This has for instance been directly demonstrated for the enzymes α-1,3-N-acetylgalactosaminyltransferase, α-1,3-galactosyltransferase, β-1,3-glucuronosyltransferase, and β-1,4-galactosyltransferase among others [43–45]. Interestingly, the glycosylation defects observed in TMEM165-CDG patients mainly correspond to altered activity of the Golgi β-1,4-galactosyltransferase that requires Mn^2+^ cations as cofactor for proper activity [17]. Therefore, the glycosylation defects observed in TMEM165-CDG patients might arise from Golgi Mn^2+^ disturbances, which would in turn impair the enzymatic activity of this specific glycosyltransferase. Similarly, some *S. cerevisiae* glycosyltransferases also require Mn^2+^ as cofactor: Och1p, Mnn1p, Mnn2p, Mnn5p, and Mnn9p [46–49]. The glycosylation defects observed in the yeast *gdt1*Δ strain grown in the presence of Ca^2+^ actually correspond to altered activity of the Mn^2+^-dependent mannosyltransferases Mnn1p, Mnn2p, and Mnn5p [23], suggesting again that these glycosylation defects might derive from altered Mn^2+^ content within the Golgi. Nevertheless, as TMEM165 and Gdt1p both seem to transport also Ca^2+^ at the Golgi membrane, one cannot rule out the possibility that the glycosylation defects are also partly caused by a disturbed Ca^2+^ balance within the Golgi lumen (and/or cytosol). Indeed, Ca^2+^ cations are essential for proper vesicular trafficking through their role in membrane fusion, and for activity and stability of enzymes involved in glycosylation [50–52]. Apart from an implication in glycosylation, transport of Ca^2+^ by Gdt1p most likely modulates cellular Ca^2+^-induced signaling in yeast (since the modulation of the cytosolic Ca^2+^ concentration observed after a salt stress is affected in case of deletion of *GDT1*) [19]. Finally, transport of Ca^2+^ and/or Mn^2+^ cations in the Golgi lumen by the UPF0016 members might constitute a detoxification pathway to prevent toxic accumulation in the cytosol in case of cation excess. Indeed, once inside the Golgi lumen, these ions can be directed out of the cell through the secretory pathway.

Apart from providing the secretory pathway with Ca^2+^ and Mn^2+^, TMEM165 and Gdt1p are also suggested to modulate the pH homeostasis of the Golgi. According to this latter statement, Gdt1p would be a putative actor in eliminating the proton load generated by glycosylation, in exchange of transport of divalent cations from the cytosol to the Golgi [28]. In the same line, the increased expression of *TMEM165* during lactation might reflect its role in providing the Golgi lumen of lactating mammary cells with Ca^2+^ as nutrient and Mn^2+^ as cofactor of enzymes, but also in removing the protons generated as by-product of lactose production out of the Golgi lumen [26, 53]. Since there are no clear proton export mechanisms from the Golgi to now both in yeast and human, this involvement of the UPF0016 members in Golgi pH homeostasis appears as highly interesting for further investigation in the near future.

Before the characterization of the plant chloroplast UPF0016 members CMT1 and PAM71, the mechanism by which Mn^2+^ and Ca^2+^ cations were delivered into the chloroplast lumen remained elusive. The current model for Mn^2+^, and putatively for Ca^2+^ delivery in the chloroplast of *A. thaliana* is that these cations would first be transported from the cytosol into the chloroplast stroma through the inner envelope CMT1, and further transferred to the thylakoid lumen by PAM71 [7]. The photosynthetic defects observed in case of malfunction of these proteins most likely arise from disturbed Mn^2+^ content within the chloroplasts. Indeed, Mn^2+^ is a structural component of the PSII. In photosynthetic organisms, the PSII mediates splitting of water into oxygen, protons, and electrons. While O_2_ is released, the PSII thereby provides electrons for further photosynthetic reactions and generates a proton gradient used by the ATP synthase to generate ATP. Affected function of the PSII therefore leads to less reducing power and ATP synthesis [54], which in turn affects plant growth and development, as observed in the UPF0016 mutant plants (reviewed in [55]). In addition, both PAM71 and CMT1 most likely play a role in maintaining proper activity of Mn^2+^-requiring enzymes in the plastid stroma. As illustration, Mn^2+^ cations are known to activate the rubisco in the chloroplasts [56]. Besides, CMT1 and PAM71 influence Ca^2+^ homeostasis from their localization at the chloroplast, which could in turn also impair photosynthesis. Indeed, Ca^2+^ is a regulator of chloroplast enzymes, and proper modulation of the Ca^2+^ concentration within the chloroplast is essential for signaling during photosynthesis [4]. On the other hand, *A. thaliana* additionally possesses two ER-localized (PML4 and PML5) and one Golgi-localized (PML3) UPF0016 members [2]. The putative role in glycosylation of these UPF0016 members at the plant secretory pathway still remains to be investigated, so are their substrate specificity and their overall role in plant cell physiology.

Since the subcellular localization of the cyanobacterial SynPAM71 is still under debate (both at the plasma membrane and thylakoid membranes, suggesting dual targeting, according to [13], or only at the thylakoid membrane according to [14]), its physiological role has to be examined in the light of these two possibilities. At the thylakoid membrane, SynPAM71 would have similar functions than in plants, i.e. contributing to Mn^2+^ supply to the Mn_4_CaO_5_ oxygen-evolution center, but also in sequestering Mn^2+^ within the thylakoid lumen to prevent toxic accumulation in the cytosol. At the plasma membrane, this protein would balance the otherwise harmful effects of Mn^2+^ accumulation in the cytoplasm, hence preventing Mn^2+^ toxicity. In this context, the photosynthetic deficiency observed in the mutant line would derive from reduced Mn^2+^ bioavailability for the PSII, and/or from toxic cytosolic Mn^2+^ accumulation [13, 14]. Determination of the exact subcellular localization of SynPAM71 thereby appears as essential to determine its physiological role.

Finally, in bacteria, the UPF0016 members would help maintaining physiological Mn^2+^ concentrations within the cytosol from their localization at the cell membrane, most likely with a Mn^2+^-dependent regulation to cope with a wide range of environmental stresses. Besides, Mn^2+^ is known to be essential for virulence of pathogenic bacteria [57]. Indeed, due to the requirement of Mn^2+^-dependent enzymes for virulence, bacteria must possess functional and well-regulated Mn^2+^ transporters. Maintenance of bacterial Mn^2+^ homeostasis is even more important in the light of the fact that mammalian host cells have developed strategies to sequester Mn^2+^ from bacterial invaders as a defense mechanism [57, 58]. Therefore, it would be highly interesting to investigate the importance of the bacterial UPF0016 members during interactions with eukaryotic hosts for example.

## STRUCTURE-FUNCITON RELATIONSHIP OF THE UPF0016 MEMBERS

**Figure 3:**
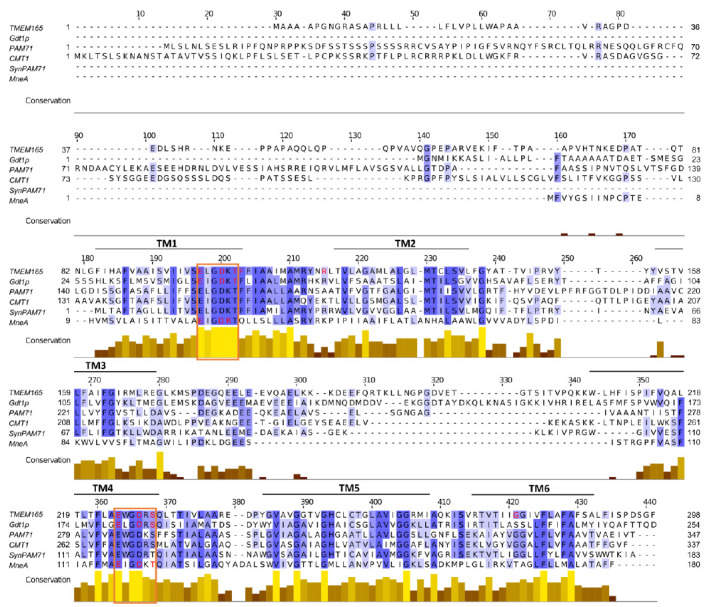
FIGURE 3: Multiple alignment of the amino acid sequences of the studied UPF0016 members. The alignment was carried out using Clustal Omega and visualized in Jalview. Residues are shaded in blue depending on their conservation. The conservation histogram provides quantitative evaluation of the degree of conservation of the physico-chemical properties of the amino acids at the corresponding position. Putative transmembrane spans predicted by TMAP are indicated above the sequences (TM1 – TM6). The highly conserved UPF0016 motifs are framed in orange. Residues identified as altering the protein stability, localization, and/or activity are highlighted in red.

Alignment of the protein sequences of the UPF0016 members reveals that the most conserved regions are the two copies of the featured motif Glu-ϕ-Gly-Asp-(Arg/Lys)-(Ser/Thr) (**Figure 3**). Therefore, the structure-function relationship of the UPF0016 members was analyzed mainly in terms of importance of the residues that constitute the two motifs to evaluate their putative role in ion coordination, but also of the residues that are found mutated in the TMEM165-CDG patients to gain insight into the mechanism of pathogenicity. **Table 1** provides an overview of the mutations that have an effect on protein function, stability, or subcellular localization.

First, due to the presence of negatively charged (Asp and Glu), positively charged (Lys or Arg), and hydroxyl-containing residues (Thr or Ser) within the UPF0016 motif predicted to be incorporated in hydrophobic transmembrane spans, these two copies were perceived as good candidates for the formation of the pore for cation transport. In yeast, production of Gdt1p with mutations in Ala of the acidic (^53^Glu & ^204^Glu, and ^56^Asp & ^207^Asp) and polar uncharged (^58^Thr and ^209^Ser) residues of the motifs in the *gdt1Δpmr1*Δ strain, that is sensitive to high Ca^2+^ concentrations and shows impaired Ca^2+^ response to a salt stress, failed to suppress these defects, whereas non-mutated Gdt1p and the other mutated versions from the motif did [59]. In the same line, expression of the mutated versions of *GDT1* coding for proteins with mutation of the acidic residues of the conserved motifs (^53^Glu & ^204^Glu, and ^56^Asp & ^207^Asp) in the *gdt1*Δ strain fails to suppress the glycosylation defects observed in the presence of high Ca^2+^ concentration [23]. Altogether, this suggests that these residues are essential for the Ca^2+^ transport activity of Gdt1p (given that these mutated proteins are produced and well localized as verified by [59]). In human, the ability of wild-type or mutated TMEM165 to rescue glycosylation defects observed in TMEM165-KO cells was similarly used to investigate the involvement of the residues of the conserved motifs, as well as of flanking residues, in protein functionality [29]. Interestingly, the mutated proteins from the motifs ^248^Glu, ^111^Asp & ^251^Asp, ^113^Thr, and ^253^Ser, as well as from the flanking residue ^114^Phe, were all unable to restore glycosylation. While none of these mutations affected protein stability, the mutated proteins ^251^Asp and ^253^Ser were found mislocalized in vesicular structures throughout the cytoplasm. Finally, the TMEM165 mutated proteins from the motifs ^108^Glu, ^111^Asp & ^251^Asp, ^113^Thr, and ^253^Ser are resistant to Mn^2+^-induced degradation, in contrast to the wild-type TMEM165 that is known to be degraded in the presence of high Mn^2+^ concentrations [29]. These data clearly illustrate the role of the acidic and polar uncharged residues of the conserved motifs in activity, localization, and Mn^2+^-induced sensitivity of TMEM165. Finally, the mutated forms ^19^Glu & ^116^Glu, ^22^Asp & ^119^Asp, ^23^Lys, and ^121^Thr in Ala from the conserved motifs of the bacterial MneA are unable to complement the Mn^2+^ sensitivity of an *E. coli* mutant strain devoid of its Mn^2+^ exported MntP [16], thereby illustrating their key importance for proper protein function in Mn^2+^ homeostasis. Taken together, study of the primary structure-function relationship of the UPF0016 members from yeast, human, and bacteria illustrates a conserved importance of the residues that constitute the UPF0016 motifs, especially of the acidic and polar uncharged ones, for protein function, as well as an additional role in protein localization and Mn^2+^ sensitivity in human. More advanced structural analyses are, however, required to confirm the implication of these residues in cation coordination at the predicted pore of the transporter.

**TABLE 1.** Summary of the mutations within the UPF0016 members MneA (*V. cholerae*), Gdt1p (*S. cerevisiae*), and TMEM165 (human) that have an effect on their transport function, stability, or subcellular localization.

**Mutation**	**Effect of the mutation**	**Ref.**
***V. cholerae* MneA (Mn^2+^ transporter)**		
^19^Glu>Ala	Altered transport activity	[16]
^22^Asp>Ala	Altered transport activity	[16]
^23^Lys>Ala	Altered transport activity	[16]
^116^Glu>Ala	Altered transport activity	[16]
^119^Asp>Ala	Altered transport activity	[16]
^121^Thr>Ala	Altered transport activity	[16]
***S. cerevisae* Gdt1p (Ca**^**2+**^ **& Mn**^**2+**^ **transporter)**		
^53^Glu>Ala	Altered transport activity	[59]
^53^Glu>Gly		[23]
^56^Asp>Ala	Altered transport activity	[59]
^56^Asp>Gly		[23]
^58^Thr>Ala	Altered transport activity	[59]
^204^Glu>Ala	Altered transport activity	[59]
^204^Glu>Gly		[23]
^207^Asp>Ala	Altered transport activity	[59]
^207^Asp>Ala		[23]
^209^Ser>Ala	Altered transport activity	[59]
**Human TMEM165 (Ca^2+^ & Mn^2+^ transporter)**		
^108^Glu>Gly	Altered transport activity Reduced Mn^2+^ sensitivity	[21, 29]
^111^Asp>Gly	Altered transport activity Reduced Mn^2+^ sensitivity	[29]
^113^Thr>Gly	Altered transport activity Reduced Mn^2+^ sensitivity	[29]
^114^Phe>Gly	Altered transport activity	[29]
^126^Arg>His	Altered subcellular localization	[60]
^126^Arg>Cys		[60]
^248^Glu>Gly	Altered transport activity	[29]
^251^Asp>Gly	Altered transport activity Reduced Mn^2+^ sensitivity Altered subcellular localization	[29]
^253^Ser>Gly	Altered transport activity Reduced Mn^2+^ sensitivity Altered subcellular localization	[29]
^304^Gly>Arg	Altered subcellular localization	[60]

The effect of the naturally-occurring mutations among TMEM165-CDG patients were also examined in terms of protein localization, expression, and activity. In this context, the mutation c.792+182 G>A (that activates a cryptic splice site) was the only one identified as conferring protein instability [60]. In contrast, the mutations ^126^Arg>His, ^126^Arg>Cys, and ^304^Gly>Arg affect the Golgi subcellular localization of TMEM165. Interestingly, the mutations corresponding to ^126^Arg>His and ^126^Arg>Cys were also introduced in *GDT1* and expressed in the *gdt1*Δ yeast strain (no residue equivalent to ^304^Gly being found in the yeast Gdt1p). Since their expression restores the ability of *gdt1*Δ to grow in Ca^2+^-rich medium, these proteins seem to be functional, thereby suggesting that the pathogenicity of these mutations is linked to altered localization rather than deficient protein activity [60]. More recently, due to optimization of the production of TMEM165 in the yeast *gdt1*Δ strain, its mutated versions were produced in this host. Using this yeast model of the disease, combined with transport assays carried out in TMEM165-producing *L. lactis* cells loaded with the fluorescent probe Fura-2, it appears that the mutation ^108^Glu>Gly leads to decreased protein activity while such reduced activity is not clear for the other mutants [21]. Altogether, these results illustrate that the pathogenicity of the mutations arises from distinct mechanisms than can now be unraveled through the biochemical tools aforementioned: altered expression (c.792+182 G>A), altered localization (^126^Arg>His, ^126^Arg>Cys, and ^304^Gly>Arg), or altered transport activity (^108^Glu>Gly) [21, 60].

## CONCLUSION

Molecular characterization of the UPF0016 members tremendously increased since their identification in 2012. The implication of the UPF0016 members in regulating cation homeostasis, thereby influencing various essential processes, is now well established in diverse organisms. For this reason, the UPF0016 family has been recently renamed as the Gdt1 family, according to the name of the yeast ortholog whose study significantly contributed to the characterization of this protein family (UniProt). Nevertheless, several aspects related to the UPF0016 family still remain to be investigated. Development of direct transport assays for the UPF0016 members in their endogenous host or proteoliposomes appears as one of the key approaches to confirm the identity of the transported substrates and their direction of transport. High-resolution structural analysis of the UPF0016 members also appears as essential for a better understanding of their mode of action.

Besides, recent results suggest that the spectrum of action of the Gdt1 family could be broader than what is currently known. Indeed, TMEM165 was found overexpressed in hepatocellular carcinoma and involved in cancer invasive activity, through a mechanism that still needs to be uncovered [61]. Another example lies in the recent identification of TMEM165 splice variants that localize at the ER, rather than at the Golgi, which also raises the possibility of additional function of TMEM165 that have not been identified yet [62]. Further studies of the Gdt1 family members will therefore most likely unravel new aspects on this captivating family of secondary transporters.

## Abbreviations:

CDG: – congenital disorder of glycosylation,
ER: – endoplasmic reticulum,
PSII: – photosystem II,
UPF: – uncharacterized protein family.
